# Supplementary material for: Resolving the full spectrum of human genome variation using Linked-Reads
Source: Genome Res. 2019 Apr;29(4):635–45. doi: 10.1101/gr.234443.118 (PMC6442396; doi:10.1101/gr.234443.118)
Supplement: Supplemental Material [file supp_gr.234443.118_Supplemental_longranger-2.2.zip › longranger-2.2.tar.bz2!/lr2.2-public/lib/stan/cmdstan/stan/doc/api/html/agrad_2fwd_2functions_2exp2_8hpp_source.html]

Stan: src/stan/agrad/fwd/functions/exp2.hpp Source File


|  |  |
| --- | --- |
|  | Stan  2.5.0  probability, sampling & optimization |


- Main Page
- Related Pages
- Namespaces
- Classes
- Files

- File List
- File Members

All Classes Namespaces Files Functions Variables Typedefs Enumerations Enumerator Friends Macros Pages

- src
- stan
- agrad
- fwd
- functions

exp2.hpp

Go to the documentation of this file.

1 #ifndef STAN\_\_AGRAD\_\_FWD\_\_FUNCTIONS\_\_EXP2\_HPP

2 #define STAN\_\_AGRAD\_\_FWD\_\_FUNCTIONS\_\_EXP2\_HPP

3

4 #include <stan/agrad/fwd/fvar.hpp>

5 #include <stan/meta/traits.hpp>

6 #include <stan/math/functions/exp2.hpp>

7 #include <stan/math/constants.hpp>

8

9 namespace stan {

10

11  namespace agrad {

12

13  template <typename T>

14  inline

15  fvar<T>

16  exp2(const fvar<T>& x) {

17  using stan::math::exp2;

18  using std::log;

19  return fvar<T>(exp2(x.val\_), x.d\_ \* exp2(x.val\_) \* stan::math::LOG\_2);

20  }

21  }

22 }

23 #endif

stan::math::LOG\_2

const double LOG\_2

The natural logarithm of 2, .

**Definition:** constants.hpp:32

stan::agrad::exp2

fvar< T > exp2(const fvar< T > &x)

**Definition:** exp2.hpp:16

stan::agrad::fvar

**Definition:** fvar.hpp:13

exp2.hpp

traits.hpp

fvar.hpp

stan::math::exp2

boost::math::tools::promote\_args< T >::type exp2(const T y)

Return the exponent base 2 of the specified argument (C99).

**Definition:** exp2.hpp:23

stan::agrad::fvar::d\_

T d\_

**Definition:** fvar.hpp:16

constants.hpp

stan::agrad::log

fvar< T > log(const fvar< T > &x)

**Definition:** log.hpp:15

stan::agrad::fvar::val\_

T val\_

**Definition:** fvar.hpp:15


---

[ Stan Home Page ]

*© 2011–2014,
Stan Development Team.*
